# Increased Atherothrombotic Burden in Patients with Diabetes Mellitus and Acute Coronary Syndrome: A Review of Antiplatelet Therapy

**DOI:** 10.1155/2012/909154

**Published:** 2012-01-05

**Authors:** Karthik Balasubramaniam, Girish N. Viswanathan, Sally M. Marshall, Azfar G. Zaman

**Affiliations:** ^1^Institute of Cellular Medicine, Newcastle University, Newcastle upon Tyne NE2 4HH, UK; ^2^Institute of Cellular Medicine, Newcastle University, Freeman Hospital, Newcastle upon Tyne NE7 7DN, UK

## Abstract

Patients with diabetes mellitus presenting with acute coronary syndrome have a higher risk of cardiovascular complications and recurrent ischemic events when compared to nondiabetic counterparts. Different mechanisms including endothelial dysfunction, platelet hyperactivity, and abnormalities in coagulation and fibrinolysis have been implicated for this increased atherothrombotic risk. Platelets play an important role in atherogenesis and its thrombotic complications in diabetic patients with acute coronary syndrome. Hence, potent platelet inhibition is of paramount importance in order to optimise outcomes of diabetic patients with acute coronary syndrome. The aim of this paper is to provide an overview of the increased thrombotic burden in diabetes and acute coronary syndrome, the underlying pathophysiology focussing on endothelial and platelet abnormalities, currently available antiplatelet therapies, their benefits and limitations in diabetic patients, and to describe potential future therapeutic strategies to overcome these limitations.

## 1. Introduction

A PubMed (Medline) search was performed using the following terms either singly or in combination: diabetes, type 2 diabetes mellitus, cardiovascular risk, hypercoagulability, prothrombotic, acute coronary syndrome, endothelial dysfunction, antiplatelet, platelet dysfunction, aspirin, clopidogrel, and glycoprotein IIb/IIIa inhibitor. All papers relevant to platelet and endothelial abnormalities in diabetes mellitus, acute coronary syndrome, and current antiplatelet therapies were considered.

Diabetes mellitus (DM) can be described as a metabolic disorder of multiple aetiology characterised by chronic hyperglycaemia with disturbances of carbohydrate, fat, and protein metabolism resulting from defects of insulin secretion, insulin action, or a combination of both [1]. The world prevalence of diabetes among adults (aged 20–79 years) was approximately 6.4%, affecting 285 million adults in 2010 and is predicted to rise to 7.7%, affecting 439 million adults by 2030 [2]. Between 2010 and 2030, there will be a 69% increase in numbers of adults with diabetes in developing countries and a 20% increase in developed countries. Globally, diabetes is likely to be the fifth leading cause of death [3]. The most prevalent form of DM is type 2 diabetes mellitus (T2DM). Insulin resistance usually precedes the onset of T2DM and is commonly accompanied by other related metabolic abnormalities such as hyperglycaemia, dyslipidaemia, hypertension, and prothrombotic factors, all of which contribute to the increased cardiovascular risk. This condition is called metabolic syndrome [4, 5].

## 2. Diabetes and Cardiovascular Disease (CVD)

A large body of epidemiological and pathological data, documents that diabetes is an important independent risk factor for CVD in both men and women [6–8]. The incidence of CVD, including coronary artery disease (CAD), stroke and peripheral arterial disease, is two- to four-fold, greater in diabetic patients than in the general population [9]. The small vessel diabetes-specific conditions of nephropathy, retinopathy, and possibly neuropathy and cardiomyopathy also contribute. In patients with T2DM, CVD is responsible for about 70% of all causes of death [10]. CVD, particularly coronary artery disease (CAD) resulting from accelerated atherosclerosis, is the leading cause of morbidity and mortality in patients with T2DM. These patients also have a higher risk of cardiovascular complications and recurrent atherothrombotic events after an index event than non-DM patients. Premenopausal women with diabetes seem to lose most of their inherent protection against developing CVD [11]. To make matters worse, when patients with diabetes develop clinical CVD, they have a poorer prognosis than the CVD patients without diabetes [12–14]. Cardiovascular mortality in patients with DM without a history of prior MI is comparable to mortality in nondiabetic subjects with previous MI [9]. Hence, diabetes has been classified as a coronary “risk equivalent” [15].

Hyperglycaemia may play an important role in increased atherothrombotic risk in DM patients. This has been supported by the Diabetes Mellitus, Insulin Glucose Infusion in Acute Myocardial Infarction (DIGAMI) trial. In this study, acute intensive glucose lowering therapy with insulin-glucose infusion led to a reduction in mortality after 3.4 years followup in DM patients with acute myocardial infarction [16]. However, in longstanding T2DM patients, chronic excessive glucose lowering (glycated haemoglobin <6.0%) was associated with increased mortality in the Action of Control Cardiovascular Risk in Diabetes (ACCORD) study [17]. This was supported by ADVANCE trial and VADT trial [18, 19]. 

## 3. Diabetes and Acute Coronary Syndrome (ACS)

Diabetes not only increases the risk of myocardial infarction (MI) but also increases the mortality associated with the acute event. The presence of DM is a strong independent predictor of short-term and long-term recurrent ischaemic events, including mortality, in patients with acute coronary syndrome (ACS). Studies have demonstrated poorer outcomes among patients with diabetes following ACS. For example, the 7-year incidence of recurrent MI in a large population-based study was 45% in diabetic patients versus 19% in nondiabetic patients. Cardiovascular mortality during that period was 42.0% and 15.4% in DM patients with and without history of acute MI, respectively [9]. The prognosis for DM patients who undergo coronary revascularisation procedures is worse than that for nondiabetic subjects [20]; DM patients experience more postprocedural complications and have decreased infarct-free survival [21]. Mortality rates for DM patients with acute MI are 1.5–2.0 times those of non-DM patients. In-hospital and 6-month mortality rates after an acute MI are highest among DM patients receiving insulin therapy. The negative impact of DM on the outcomes is maintained across the ACS spectrum, including unstable angina and non-ST elevation myocardial infarction (NSTEMI) [22], ST elevation myocardial infarction (STEMI) treated medically [23], and ACS undergoing percutaneous coronary intervention (PCI) [24]. DM patients have more progressive, diffuse, and multivessel coronary disease compared to nondiabetic patients and have poorer outcomes after both PCI (especially with bare metal stent [BMS]) and coronary artery bypass graft surgery (CABG), compared to nondiabetic patients [25]. The advent of drug eluting stents (DES) has improved PCI outcomes but the problem of atherothrombotic complications, including stent thrombosis, persists in diabetic patients [26].

The majority of acute CV events are precipitated by vascular occlusion caused by atherosclerotic plaque disruption, platelet aggregation, platelet adhesion, and the resulting intravascular thrombosis. Systems that are involved in maintaining the integrity and patency of the vasculature including endothelial and platelet function, coagulation, and fibrinolysis are impaired in diabetes, thereby shifting the balance to favour thrombus formation.

## 4. Endothelial Dysfunction and Inflammation in Diabetes

Endothelial dysfunction (ED) plays an important role in the pathogenesis and clinical expression of atherosclerosis. It has been linked to T2DM and insulin resistance states such as obesity in experimental and clinical studies [27]. ED refers to an impairment of the ability of the endothelium to properly maintain vascular homeostasis. This reflects a number of abnormalities that include loss of bioavailable nitric oxide (NO), increased production of vasoconstrictors, and disturbed regulation of inflammation, thrombosis, and cell growth in the vascular wall [28, 29]. The endothelium plays a key role in the regulation of blood flow and arterial tone by orchestrating the production of vasodilators such as NO, prostacyclin (PGI_2_) and endothelium derived hyperpolarising factor (EDHF), and vasoconstrictors including endothelin-1 (ET-1) and angiotensin II [28]. Vasodilators oppose the effects of vasoconstrictors and act in a homeostatic fashion to maintain normal arterial compliance and patency.

The contribution of inflammation to the pathogenesis of atherosclerosis is well recognised [30]. NO, which is produced by a family of enzymes called endothelial NO synthase (eNOS), not only causes vasodilatation but also prevents leukocyte adhesion and maintains the endothelium in a quiescent, anti-inflammatory state [28]. In the presence of risk factors such as DM (hyperglycaemia), endothelial cells are activated to express adhesion molecules such as vascular cell adhesion molecule-1 (VCAM-1) and intercellular adhesion molecule-1 (ICAM-1) which are required for leukocyte adhesion to the endothelial surface [31]. Endothelial expression of chemotactic factors such as monocyte chemo-attractant protein-1 and other proinflammatory cytokines like macrophage colony stimulating factor and tumour necrosis factor-beta (TNF-*β*) contributes to the inflammation within the vasculature and promotes atherogenesis [31, 32]. Endothelial production of prothrombotic molecules [plasminogen activator inhibitor-1 (PAI-1), thromboxane, tissue factor (TF) and von Willibrand's factor (vWF)] is balanced by the production of antithrombotic molecules such as NO, heparins, prostacyclin, tissue plasminogen activator and thrombomodulin. Risk factors including DM shift the balance towards a prothrombotic, antifibrinolytic state (Table 1). Selective impairment of phosphoinositide-3 kinase (PI3 kinase)/Akt kinase signalling characterises insulin resistance contributing to ED in T2DM [33]. Nicotinamide adenine dinucleotide phosphate-oxidase (NADPH oxidase) generates superoxide anion in inflammatory cells and is also involved in normal cell signalling in endothelial cells [34]. In DM, NADPH oxidase activity and superoxide production are increased [35, 36]. This increased oxidative stress impairs NO bioavailability leading to ED. NADPH oxidase promotes the activation of the proinflammatory transcription factor [nuclear factor kappa B (NF*κ*B)] [37]. Angiotensin II upregulates NADPH oxidase expression, and this could be one of the reasons for angiotensin converting enzyme inhibitors (ACEIs) having favourable vascular effects in DM [38–40].

DM is associated with a systemic inflammatory state that may contribute to ED and atherosclerosis [41]. Increased glucose and free fatty acid concentrations has been shown to activate the endothelium in various experimental studies [37, 42, 43]. Patients with DM or obesity have increased circulating levels of inflammatory markers including C-reactive protein (CRP), tumour necrosis factor-alpha (TNF-*α*), interleukin-6 (IL-6), and ICAM-1 [44–47]. Furthermore, increased levels of circulating inflammatory markers predict cardiovascular risk in DM patients [48].

Protein kinase C beta (PKC*β*) activation may also explain the link between inflammation, ED, and insulin resistance in DM [49]. PKCs are a type of serine/threonine kinase that act at the plasma membrane in the regulation of signal transduction in various cell types. The PKC*β* isoform in the endothelial cells is activated by diacylglycerol (DAG) under the conditions of increased glucose and fatty acid concentrations [50, 51]. PKC*β* reduces eNOS phosphorylation/activation by inhibiting PI3 kinase and Akt and also activates NF*κ*B [52–55]. In humans, acute treatment with a PKC*β* inhibitor prevented the development of ED following glucose infusion in the forearm of healthy volunteers and improved brachial artery flow-mediated dilatation in DM patients [56, 57].

DM and insulin resistance are strongly linked to abnormalities of mitochondrial function [58–60]. DM impairs mitochondrial biogenesis, fusion, and autophagy, leading to cells with decreased mitochondrial mass and a predominance of fragmented and dysfunctional mitochondria [60, 61]. This leads to increased amounts of reactive oxygen species and decreased amounts of ATP. Impaired mitochondrial energetics leads to increased levels of DAG which activates PKC*β* and this in turn impairs NO production. Recent work has demonstrated links between ED, impaired mitochondrial biogenesis, and increased mitochondrial superoxide production in arterioles isolated from patient with DM compared to healthy controls [62].

## 5. Platelets in Diabetes: “Angry Diabetic”  Platelets

ACS is precipitated by the ischaemic effect of an occlusive intracoronary thrombus that develops over a ruptured atheromatous plaque as a result of platelet adhesion and aggregation [63]. Among diabetic individuals increased platelet aggregation and adhesion are due to the following.

- Reduced platelet membrane fluidity due to changes in the lipid composition of the membrane or glycation of membrane proteins.
- Increased production of thromboxane A_2_ (TXA_2_) from arachidonic acid metabolism, which increases platelet sensitivity [64].
- Increased expression of platelet adhesion molecules such as CD31, CD36, CD49b, CD62P, and CD63 (when assessed by flow cytometry) [65].
- Upregulation of platelet ADP P2Y_12_ receptor signalling, which suppresses cAMP levels and lowers insulin responsiveness, thereby leading to increased adhesion, aggregation and pro-coagulant activity.
- Increased expression of platelet surface receptors such as P-selectin, Glycoprotein (GP) Ib, and GP IIb/IIIa [66]. GP Ib mediates platelet binding to vWF, an important step in platelet dependent thrombosis, and GP IIb/IIIa binds to fibrinogen resulting in platelet cross-linking as a part of the aggregation process [67].
- Increased generation of platelet dependent thrombin.
- Platelets are less sensitive to the effects of PGI_2_ and NO [68].
- Platelets in T2DM show disordered calcium and magnesium haemostasis. Intraplatelet calcium regulates a variety of activities including platelet shape change, secretion, aggregation, and TXA_2_ formation [41, 69]. Increased intracellular calcium and decreased intracellular magnesium has been linked to platelet hyperaggregability and adhesiveness [70].
- Active platelets in DM patients are also rich in cytokines and chemokines such as platelet factor-4, interleukin-1*β* and CD40L and hence contribute to inflammation and atherogenesis alongside a procoagulant milieu [30, 71–73].
- Accelerated platelet turnover resulting in increased reticulated platelets also contributes to platelet hyperactivity [74].

These characteristics may play a role not only in the higher risk of developing ACS with poorer outcomes observed in DM, but also in the large proportion of DM patients with inadequate response to antiplatelet agents compared to non-DM patients. This in itself may contribute to the poorer outcomes observed in DM patients despite compliance with the recommended secondary prevention therapy with antiplatelet agents.

## 6. Microparticles in Diabetes

Besides platelets, microparticles (MPs) are also involved in diabetic atherothrombosis. MPs are membrane-coated vesicles that emerge by budding from their parental cells upon activation or apoptosis [75]. They retain at least some functions of their cells of origin, which can include platelets, endothelial cells, and various leukocytes. MPs have the ability to activate the coagulation cascade with consequent thrombosis formation [76]. Platelet-derived MPs (PMPs) expose negatively charged phospholipids which act as binding sites for activated coagulation factors [77]. Platelet MPs also bind to the subendothelial matrix and act as a substrate for further platelet adhesion via GP IIb/IIIa fibrinogen bridging [78]. Resting platelets express pre-mRNA for TF, and this gets converted to mature mRNA upon platelet activation [79]. This may allow platelets to synthesize active TF for thrombus propagation and stabilization. Monocyte-derived MPs (MMPs) exposing TF have P-selectin glycoprotein ligand-1 which interacts with P-selectin on the surface of activated platelets and help in further stabilization of thrombus [78]. Other possible pathways regulated by MPs include production of lysophosphatidic acid (a strong platelet agonist), endothelial and leukocyte activation, recruitment of monocyte within the plaque, stimulation of neoangiogenesis, induction of apoptosis in endothelial or smooth muscle cells, and increase in TXA_2_ release which in turn causes vasoconstriction [80, 81]. Increased levels of platelet-derived MPs and their role in macrovascular complications have been reported in DM patients [82]. Elevated levels of TF-positive MPs have been found to correlate with components of metabolic syndrome in patients with uncomplicated T2DM [83, 84]. Levels of PMPs and MMPs have been shown to correlate with DM complications like diabetic retinopathy, which is associated with microvascular damage [85]. Elevated endothelial cell-derived MPs (EMPs) are predictive for the presence of coronary artery lesions, and are a more significant independent risk factor than the duration of DM, lipid levels, and history of hypertension [86]. In patients with T2DM and ACS, elevated EMPs have been linked to noncalcified atheromatous lesions as detected by multidetector computed tomography [87]. Increased levels of procoagulant TF-positive MPs were demonstrated within the occluded coronary artery of patients with STEMI [88]. Insulin has been found to reduce the expression of TF in monocytes and MMPs [89]. Beneficial effects of statins in T2DM and atherothrombosis are possibly due to its effects on MPs [90–93]. All this evidence indicates that MPs are not only a reliable marker for vascular injury but they also actively participate in promoting atherothrombotic complications in T2DM [76]. In this context, drugs that may reduce the release of MPs and/or their thrombogenicity may have the potential to improve the effects of current antiplatelet therapy, resulting in lower adverse event rates in DM patients.

## 7. Platelet Leukocyte Aggregates (PLAs) in Diabetes

Platelets and leukocytes from DM patients are hyperreactive and express more adhesion molecules. P-selectin is one of the markers of platelet activation and is the main link for platelet adhesion to circulating leukocytes [94]. Platelet P-selectin interacts with leukocyte P-selectin glycoprotein ligand (PSGL-1) leading to the formation of PLA [95, 96]. Consequently, activated leukocytes secrete several pro-inflammatory cytokines and express a prothrombotic membrane phenotype. Elevated levels of circulating PLA have been reported in various prothrombotic states including DM thereby suggesting their involvement in the pathogenesis of atherothrombosis. Elevated circulating PLA levels were found to be linked with vascular injury in DM patients [94]. A significant increase in circulating platelet-polymophonuclear aggregates (PPA) and platelet-monocyte aggregates (PMAs) percentages was demonstrated in DM patients [97]. Interestingly, percentages of circulating PPA, and PMA were significantly higher in DM with vascular injury compared to DM without vascular injury suggesting the involvement of inflammatory leukocytes in vascular damage [97, 98]. Increased circulating PMA percentage was found to be more specifically a marker of diabetic microretinopathy [97]. This reinforces the fact that pro-inflammatory cells are involved in microvascular complications in DM and atherothrombotic process. Circulating PLA determination using flow cytometry may be used as a simple marker of microvascular injury in DM patients.

## 8. Coagulation in Diabetes

Tissue Factor and Factor VII (F VII) initiate the thrombotic process, resulting in the generation of thrombin. This subsequently helps in the conversion of fibrinogen into a three-dimensional network of fibrin fibres which forms the skeleton of the blood clot [99]. TF is an integral prothrombotic transmembrane protein expressed by both vascular and nonvascular cells, including monocytes, macrophages, and platelets [100]. In T2DM, TF expression is upregulated due to the presence of low-grade inflammation [100]. Levels of TF in atherosclerotic plaques in patients with unstable angina are higher compared with those who have stable angina [101]. Plasma TF levels are raised in subjects with CAD particularly ACS further emphasising the role of TF in atherothrombosis [102, 103]. Patients with T2DM have higher circulating TF levels which are directly modulated by glucose and insulin, and the two appear to have an additive effect [104] (Table 2). In T2DM, increased levels of advanced glycation end products and reactive oxygen species activate NF*κ*B which in turn leads to increased TF production [100].

F VII is a vitamin K dependent coagulation factor synthesised in the liver. F VII coagulant activity (F VII: c) has been associated with fatal cardiovascular events [105–107]. T2DM subjects have elevated F VII:c levels [108]. An association between triglyceride levels and F VII:c levels has been demonstrated; this appears to be independent of obesity and insulin resistance [109].

Thrombin concentration influences fibrin clot formation and also determines the clot structure and stability [110]. High thrombin concentration results in denser and less permeable clots which are more resistant to lysis. Thrombin generation is enhanced in DM secondary to low-grade coagulation system activation [104, 111].

Plasma fibrinogen is a well known independent CVD risk factor and is used as a surrogate marker for CVD risk [112]. High fibrinogen levels predict silent myocardial ischaemia in T2DM patients [113]. High levels of IL-6 in diabetes stimulate fibrinogen synthesis by the hepatocytes, representing a link between inflammation and the prothrombotic state [114]. Insulin resistance is also associated with increased hepatocyte fibrinogen synthesis [115, 116].

## 9. Fibrinolysis in Diabetes

Fibrinolysis is initiated by the conversion of plasminogen to plasmin, and this is largely mediated by tissue plasminogen activator (tPA). Plasminogen activator inhibitor-1 (PAI-1) is the main inhibitor of fibrinolysis by binding to tPA and forming PAI-1/tPA complex. In a long-term 18-year study, glycated haemoglobin (HbA_1C_) correlated positively with PAI-1 and negatively with tPA. This implicates hyperglycaemia as a reason for elevated PAI-1 levels [117]. Hyperinsulinaemia also has been shown to increase PAI-1 levels, which may account for elevated PAI-1 levels in insulin-resistant states [118, 119] (Table 2).

A study indicated that clots derived from plasma purified fibrinogen in 150 diabetic subjects had a more compact structure characterised by smaller pore size, increased fibrin thickness, and number of branch points than that from 50 healthy controls [120]. Clot lysis from diabetic patients was slower when compared to controls due to elevated PAI-1 levels.

## 10. Platelet Dependent Thrombus Formation

**Figure 1:**
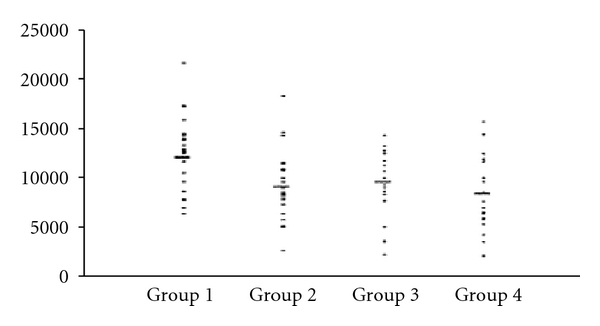
Graph representing area of thrombus along *y* axis (*μ* ^2^/mm) Group 1: T2DM with CAD; Group 2: T2DM without clinical macrovascular disease; Group 3: CAD without DM; Group 4: Healthy Controls. Adapted from [122].

Arterial injury models which simulate in vivo coronary artery rheology have been used in various experimental studies to measure platelet-dependent thrombus formation. These provide a measure of actual thrombus which is the end point of all haemostatic functions. Work from our laboratory using the arterial injury model demonstrated increased blood thrombogenicity and platelet dependent thrombus formation in patients with T2DM and CAD, compared to patients with T2DM without CAD and nondiabetic controls, despite being on aspirin and other evidence based risk factor therapy (Figure 1) [121, 122]. An improvement in blood thrombogenicity in diabetic subjects with improved glycaemic control has been shown [123]. In another study, we compared blood thrombogenicity and clot kinetics in 30 T2DM and 30 nondiabetic patients, one week after troponin positive non-ST elevation ACS (NSTE-ACS). In patients with T2DM, the thrombus was increased in quantity [area of thrombus: mean (SD) [16824.0(3619.1) versus 14413.9(4648.5) *μ* ^2^/mm, *P* = 0.029], weaker in tensile strength [clot index: median (range) 0.4 (−3.3 to 4.2) versus 1.6 (−1.5to 5.4), *P* = 0.026] and more importantly resistant to clot retraction [clot lysis: median (range) 27.7 (3.8–83.4) versus 73.7 (55–191) mm/min, *P* = 0.017. This was despite the participants being prescribed currently recommended secondary prevention therapy including aspirin and clopidogrel, after a NSTE-ACS [124]. These findings may partly explain the reason for reduced benefits of current antiplatelet therapy in patients with diabetes [125]. 

## 11. Antiplatelet Therapies in Diabetes

**Figure 2:**
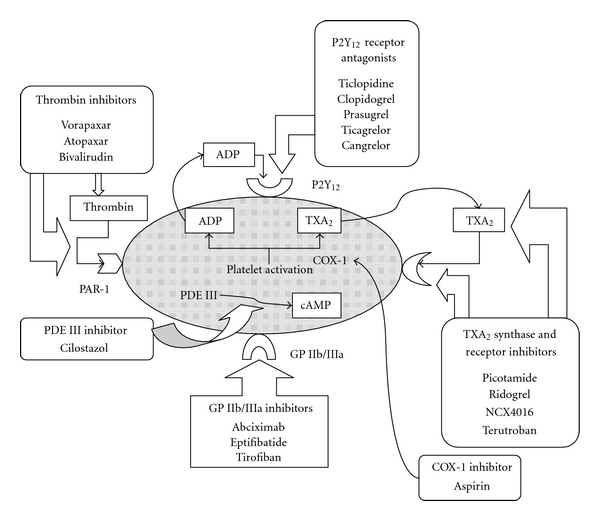
Schematic representation of mechanisms of action of antiplatelet agents. PAR-l: protease activated thrombin receptor-1. Adapted from [128].

A clear benefit of antiplatelet agents in the prevention of atherothrombotic events in those at high risk is well established. Multiple genetic, iatrogenic, and environmental factors influence platelet responsiveness to these agents. Three different classes of antiplatelet agents are approved for treatment and/or prevention of ACS: cyclooxygenase-1 (COX-1) inhibitors (aspirin), ADP P2Y_12_ receptor antagonists (thienopyridines), and platelet GP IIb/IIIa inhibitors [126, 127] (Figure 2). 

## 12. Aspirin

Aspirin selectively acetylates the COX-1 enzyme, thereby blocking TXA_2_ synthesis in platelets. This effect is irreversible since the platelets are enucleate and, therefore, unable to resynthesise COX-1. Aspirin use for primary prevention in DM patients has been controversial [129–131], but ongoing studies including A Study of Cardiovascular Events in Diabetes (ASCEND; NCT00135226) and Aspirin and simvastatin Combination for Cardiovascular Events Prevention Trial in Diabetes (ACCEPT-D; ISRCTN48110081) will provide further insight into this area. Aspirin, however, remains the antiplatelet agent of choice for secondary prevention of recurrent ischaemic events in patients with atherothrombotic disease including those with DM [126, 127, 132–134]. Benefits of aspirin in the early management of ACS patients including unstable angina/NSTEMI [135–137] and STEMI [138, 139] have been demonstrated consistently in various studies. Aspirin should be given as early as possible at an initial dose of 162 to 325 mg followed by a daily maintenance dose of 75 to 162 mg [126, 127]. The recommended dose of aspirin for secondary prevention in DM patients with atherothrombotic disease is 75 to 162 mg [133]. Withdrawal of aspirin can lead to recurrence of ACS [140]. Two large meta-analysis trials performed by the Antiplatelet Trialists' Collaboration included 287 studies and involved 212000 high-risk patients (with acute or previous vascular disease or other predisposing condition increasing the risk of occlusive vascular disease) [141, 142]. Aspirin was the frequently used antiplatelet agent at doses ranging from 75 to 325 mg daily in these trials. The incidence of vascular events on aspirin was reduced from 22.3% to 18.5% in DM cohort (*P* < 0.002) and from 16.4% to 12.8% (*P* < 0.00001) in non-DM cohort. The incidence of vascular events was much higher in DM patients but the benefit of antiplatelet therapy was consistent regardless of the DM status [141]. Low-dose aspirin (75 to 150 mg) was as effective as higher daily doses but with significantly lower bleeding complications [141, 142]. The Clopidogrel Optimal Loading Dose Usage to Reduce Recurrent Events/Organisation to Assess Strategies in Ischaemic Syndromes (CURRENT/OASIS-7) trial is the first large-scale randomised study comparing high- and low-dose aspirin. This study randomised ACS patients who were scheduled to undergo angiography within 72 hours of hospital arrival [143, 144]. With a  2 × 2 factorial design, patients were randomised (double blinded) to high or standard dose clopidogrel for a month and in open label fashion to high-dose (300 to 325 mg daily) or low-dose (75 to 100 mg daily) aspirin. There was no significant difference in the rates of the primary outcome (cardiovascular death, MI or stroke after 30 days) between high and low dose aspirin (4.1% versus 4.2%; hazard ratio [HR] = 0.98; *P* = 0.76) in DM and non-DM patients. A trend towards higher gastrointestinal bleeding rates (0.38% versus 0.24%; *P* = 0.051) in the high dose group was observed [143].

## 13. P2Y_12_ Receptor Antagonists

Platelet ADP signalling pathways mediated by P2Y_1_ and P2Y_12_ play an important role in platelet activation and aggregation. P2Y_12_ activation leads to sustained platelet aggregation and stabilisation of the aggregated platelets [145, 146]. Thienopyridines (Ticlopidine, Clopidogrel,8 and Prasugrel) which are nondirect, irreversible P2Y_12_ antagonists are currently approved for clinical use.

Ticlopidine was the first thienopyridine that was approved for clinical use in 1991. Ticlopidine in combination with aspirin was superior to aspirin alone or anticoagulation with aspirin in various clinical trials for the prevention of recurrent ischaemic events in patients undergoing PCI [147–150]. Ticlopidine (high rates of neutropenia) was largely replaced by clopidogrel (a second generation thienopyridine) due to its better safety profile [151].

Clopidogrel is the most widely used thienopyridine and has a faster onset of action through the administration of a loading dose [152]. The Clopidogrel versus Aspirin in Patients at Risk of Ischemic Events (CAPRIE) trial compared the efficacy of clopidogrel (75 mg/day) and aspirin (325 mg/day) in reducing the risk of ischaemic outcomes in patients (*n* = 19 185) with recent MI, recent ischaemic stroke, or established peripheral arterial disease. The results showed a significantly low annual rate of ischaemic events (ischaemic stroke, MI, or vascular death) in patients who received clopidogrel (5.32% versus 5.83%; *P* = 0.043) [153]. DM subgroup had a significantly higher benefit (15.6% versus 17.7%; *P* = 0.042). For every 1000 DM patients treated with clopidogrel, 21 vascular events were prevented [154].

Several large scale trials have shown, clear benefit of clopidogrel in addition to aspirin in preventing recurrent ischaemic events, including stent thrombosis in the setting of ACS (including unstable angina/NSTEMI, STEMI, PCI), compared to aspirin alone [22, 155–159] (Table 3). Current guidelines recommend dual antiplatelet therapy with aspirin and clopidogrel for patients with ACS, including unstable angina/NSTEMI, STEMI, and patients undergoing PCI [126, 127, 134]. The recommended clopidogrel dose is 300 mg loading dose (up to 600 mg in PCI setting) followed by a maintenance dose of 75 mg daily. The Clopidogrel for High Atherothrombotic Risk and Ischemic Stabilization, Management and Avoidance (CHARISMA) trial found no benefit of dual aspirin and clopidogrel therapy in the long-term in a wide range of non-ACS patients (*n* = 15 603) with risk factors for, and established atherothrombotic disease including DM patients (*n* = 6555; 42% of the study population) [160]. Hence, dual antiplatelet therapy is not recommended even in DM patients outside ACS/PCI setting.

In the CURRENT/OASIS-7 trial, high-dose clopidogrel regimen (600 mg loading dose, then 150 mg/day for 7 days followed by 75 mg daily) significantly reduced the rates of cardiovascular death, MI, or stroke at 30 days (3.9% versus 4.5%; HR = 0.85; *P* = 0.036) as well as the risk of stent thrombosis (0.7% versus 1.3%; HR = 0.54; *P* = 0.0001) in a subgroup of patients who underwent PCI (*n* = 17 232) [143]. But among DM patients, rates of the primary outcome were not significantly different with high and standard dose clopidogrel (4.9% versus 5.6%; HR = 0.89; 95% confidence interval [CI], 0.66 to 1.15; *P* = 0.434) [144].

Prasugrel is a third generation thienopyridine that was recently approved for clinical use in ACS patients undergoing PCI. It is an orally administered, prodrug that requires hepatic metabolism to be converted to its active metabolite that irreversibly inhibits the P2Y_12_ receptor [161]. Prasugrel has a more rapid onset of action than clopidogrel with greater platelet inhibition because it is converted to its active metabolite more effectively [162]. The TRial to assess Improvement in Therapeutic Outcomes by optimizing platelet InhibitioN With prasugrel-Thrombolysis In Myocardial Infarction 38 (TRITON-TIMI 38) examined the efficacy and safety of Prasugrel (60 mg loading dose followed by 10 mg daily) versus clopidogrel (300 mg loading dose followed by 75 mg daily) in patients (*n* = 13 608) with ACS undergoing PCI [163]. A significant reduction in the rates of primary end point (consisting of cardiovascular death, nonfatal MI or nonfatal stroke) favouring prasugrel (9.9% versus 12.1%; HR = 0.81; *P* < 0.001) was found. Reduction in the rates of stent thrombosis over a follow-up period of 15 months was also found in the prasugrel treated group, but with increased risk of major bleeding [164]. The subgroups of patients with DM or with STEMI had a higher beneficial effect. In DM patients, the risk of primary end point was reduced significantly with prasugrel (12.2% versus 17%; HR = 0.70; *P* < 0.001). Prasugrel also improved the risk of stent thrombosis in DM subgroup (all DM patients: 2.0% versus 3.6%; HR = 0.52; *P* = 0.007; insulin treated patients: 1.8% versus 5.7%; HR = 0.31; *P* = 0.008) [165]. Major bleeding was higher in DM patients over all but there was no difference among DM patients treated with either prasugrel or clopidogrel (2.6% versus 2.5%; HR = 1.06; *P* = 0.81).

The Optimizing antiplatelet Therapy in diabetes MellitUS-3 (OPTIMUS-3) study showed that prasugrel (60 mg loading dose followed by 10 mg/day maintenance dose) achieved significantly greater platelet inhibition compared to double dose clopidogrel (600 mg loading dose followed by 150 mg/day maintenance dose) in DM patients with CAD. Verify Now P2Y_12_ assay demonstrated more effective platelet inhibition with prasugrel compared to clopidogrel, 4 hours after the loading dose (least squares mean, 89.3% versus 27.7%; *P* < 0.0001). This difference in platelet inhibition remained significant up to 7 days after dosing [166–168]. Higher platelet inhibition in DM patients results in greater clinical benefit as suggested by all the above observations. The clinical efficacy of prasugrel when compared to clopidogrel in medically managed patients with unstable angina/NSTEMI is being evaluated in the ongoing TaRgeted platelet Inhibition to cLarify the Optimal strateGy to medicallY manage Acute Coronary Syndrome (TRILOGY ACS) trial.

Ticagrelor is an orally administered, direct reversible P2Y_12_ inhibitor which achieves higher platelet aggregation inhibition compared to clopidogrel in ACS patients [169]. The PLATelet inhibition and patient Outcomes (PLATO) trial compared ticagrelor (180 mg loading dose followed by 90 mg twice daily) with clopidogrel (300 to 600 mg loading dose followed by 75 mg daily) in preventing cardiovascular events in ACS patients (*n* = 18 624). The rate of primary end point (death resulting from MI or stroke) at 12 months decreased significantly in patients treated with ticagrelor (10.2% versus 12.3%; HR = 0.84; *P* = 0.0001). In the PCI subgroup the rate of CV death and stent thrombosis reduced significantly. Ticagrelor was not associated with an increase in protocol defined major bleeding. In DM patients, ticagrelor reduced the rates of the primary end point (HR = 0.88), all cause mortality (HR = 0.82) and stent thrombosis (HR = 0.65) compared to clopidogrel. Similar benefits were seen in patients with or without insulin treatment. Bleeding rates were similar (HR = 0.98) in ticagrelor and clopidogrel treated DM patients. Side effects reported included dyspnoea, ventricular pauses, and increase in creatinine and uric acid levels [170, 171]. Ticagrelor has been approved for use in Europe but not yet approved for clinical use by the FDA.

## 14. GP IIb/IIIa Inhibitors

Three different GP IIb/IIIa inhibitors (abciximab, tirofiban and eptifibatide) are currently approved for clinical use. All these agents can be administered only intravenously, and hence their use is limited to acute settings. A meta-analysis of 6 large clinical trials evaluating the effect of GP IIb/IIIa inhibitors in ACS patients showed a 22% reduction of mortality at 30 days in DM patients (*n* = 6458) who received GP IIb/IIIa inhibitor compared to those who did not (4.6% versus 6.2%; *P* = 0.007). Non-DM patients (*n* = 23 072) had no survival benefit [172]. The benefit was greater among the DM patients who underwent PCI during index hospitalisation (*n* = 1279, 1.2% versus 4%; *P* = 0.002). Unfortunately, these trials were performed using ticlopidine or standard dose clopidogrel instead of the high loading dose of clopidogrel. A high loading dose of clopidogrel has a more potent antiplatelet effect and is the current recommendation for ACS patients who undergo PCI.

A recent study, the Intracoronary Stenting and Antithrombotic Regimen: Is Abciximab a Superior Way to Eliminate Elevated Thrombotic Risk in Diabetics (ISAR-SWEET) trial, compared the effect of abciximab and placebo on the 1-year risk of death and MI in DM patients (*n* = 601) who underwent elective PCI after pretreatment with 600 mg loading dose of clopidogrel at least 2 hours before the procedure [173]. There was no beneficial effect from abciximab in this study, suggesting that routine use of GP IIb/IIIa inhibitor in elective PCI (non-ACS setting) is not recommended.

In the Intracoronary Stenting and Antithrombotic Regimen: Rapid Early Action for Coronary Treatement-2 (ISAR-REACT 2); trial, abciximab was shown to be significantly beneficial in patients with high-risk ACS undergoing PCI after pretreatment with 600 mg of clopidogrel [174]. These trials support the use of GP IIb/IIIa inhibitors in high-risk ACS patients, including DM patients as recommended in the current guidelines [126].

A meta-regression analysis of randomised trials evaluated the effect of GP IIb/IIIa inhibitors in STEMI patients treated with primary PCI. These agents showed benefit in terms of death but not re-infarction in high risk patients including DM patients [175]. The major limitation of GP IIb/IIIa inhibitors is the increased risk of bleeding, and it is well known that bleeding has a significant impact on prognosis after an ACS, including mortality [176, 177].

## 15. Direct Thrombin Inhibitors

Bivalirudin, a direct thrombin inhibitor, provided similar protection from ischaemic events as GP IIb/IIIa inhibitors, but with lower bleeding rates in the Acute Catheterisation and Urgent Intervention Triage strategY (ACUITY) trial [178]. In a subgroup analysis in DM patients, rates of primary end point (death, MI or unplanned ischaemic revascularisation) were similar on bivalirudin monotherapy and GP IIb/IIIa inhibitor plus heparin treatment (7.9% versus 8.9%; *P* = 0.39). The rate of major bleeding was significantly lower in the bivalirudin group (3.7% versus 7.1%; *P* = 0.02). This bleeding risk reduction is particularly important because the risk of bleeding complications is increased in diabetic patients with ACS undergoing PCI [179].

## 16. Antiplatelet Drug Resistance in Diabetes

“Resistance” means that the antiplatelet agent fails to block its specific target (e.g., aspirin to block the COX-1 enzyme and clopidogrel to block the P2Y_12_ receptor) [180]. Aspirin resistance is associated with a higher risk of recurrent ischaemic events [181, 182]. COX-1-specific tests (urine thromboxane and assays using arachidonic acid as an agonist) and non-COX-1-specific tests are available. Poor patient compliance is the main reason for aspirin resistance when a COX-1-specific test is used [183]. DM patients respond inadequately to aspirin when assessed by non-COX-1-specific tests [184, 185]. In the Aspirin-Induced Platelet Effect (ASPECT) study, a subanalysis showed that aspirin resistance was common in DM patients while on a low dose (81 mg daily). Increasing the aspirin dose (162 and 325 mg daily) significantly reduced the platelet activity [186]. There has been no study designed to date to assess the implications of aspirin resistance in DM patients with ACS. Hyperglycaemia, increased TXA_2_ synthesis, increased platelet turnover, and TXA_2_ receptor (TP receptor) activation have all been proposed to play a key role in aspirin resistance in DM patients [187, 188].

Genetic, cellular, and clinical mechanisms contribute to inadequate clopidogrel responsiveness [189, 190]. DM is an important factor contributing to decreased clopidogrel effects. DM patients show lower response to clopidogrel both in the loading phase and in the maintenance phase when compared to non-DM patients [184, 191, 192]. DM patients who are on insulin have the highest degree of platelet reactivity while on dual antiplatelet therapy [193]. Chronic kidney disease in DM patients has been associated with impaired clopidogrel response [194]. All these findings suggest why DM is associated with higher risk of recurrent ischaemic events in patients with ACS [22] and is a strong predictor of stent thrombosis [195–197].

## 17. Future Treatment Options in Diabetic Patients with ACS

Persistent high platelet activity in DM patients despite current recommended antiplatelet therapy has raised interest in identifying strategies to optimise platelet inhibition in this high-risk population.

## 18. Dose Modification of Antiplatelet Agents

The OPTIMUS study showed marked improvement in platelet inhibition when 150 mg maintenance dose of clopidogrel was used in DM patients with CAD [167]. In the Gauging Responsiveness with A VerifyNow Assay-Impact On Thrombosis And Safety (GRAVITAS) trial, high clopidogrel dose (600 mg loading dose followed by 150 mg daily maintenance dose for 6 months) was given to patients with inadequate response to standard clopidogrel dose. At six months of followup, the composite end point of cardiovascular death/MI/stent thrombosis was identical in both groups, at 2.3% (HR = 1.01; 95% CI, 0.58–1.76; *P* = 0.97). Stent thrombosis occurred in 0.5% of the high-dose group and 0.7% of the standard-dose group, a nonsignificant difference. There was also no difference in bleeding [198]. So there is no evidence that increase in the dose clopidogrel will improve the outcomes in patients with T2DM.

## 19. Newer Agents

### 19.1. Picotamide

Picotamide is an inhibitor of both TXA_2_ synthase and TP receptor. Increased platelet turnover in DM generates new platelets that have not been exposed to aspirin and hence continue to generate TXA_2_. Therefore, TP receptors may remain activated despite COX-1 inhibition, contributing to aspirin hyporesponsiveness. This suggests that TP antagonism may potentially be the future antiplatelet target for new pharmacological agents [199]. The Drug evaluation in Atherosclerotic Vascular disease In Diabetics (DAVID) trial compared picotamide with aspirin in DM patients with peripheral arterial disease. The 2-year overall mortality was significantly lower among those treated with picotamide compared to those receiving aspirin (3.0% versus 5.5%; P=0.0474) [200]. Other newer agents such as ridogrel (TXA_2_ synthase inhibitor and TP receptor blocker), NCX 4016 (NO-releasing aspirin derivative) and terutroban (TP receptor inhibitor) have been compared with aspirin in different studies and might be of interest in the future in DM patients [201–204].

### 19.2. Cangrelor

This is an intravenous, direct acting and reversible P2Y_12_ receptor inhibitor [205]. Phase II trials showed cangrelor to be a potent antiplatelet agent achieving a greater degree of platelet inhibition (>90%) with extremely rapid onset and offset of action [206]. However, the Cangrelor versus Standard Therapy to Achieve Optimum Management of Platelet Inhibition (CHAMPION) study failed to show any superiority of cangrelor over clopidogrel [207, 208].

### 19.3. Cilostazol

This is a phosphodiesterase III (PDE III) inhibitor that increases intraplatelet cAMP concentration. This drug may be considered in the maintenance phase of therapy along with standard dual antiplatelet therapy. A reduction in the rate of target lesion revascularisation and in-stent thrombosis has been observed in patients undergoing PCI, with this triple therapy [163, 209]. The benefit is greater in DM patients [210, 211]. The OPTIMUS-2 study also demonstrated markedly increased inhibition of platelet P2Y_12_ signalling in DM patients who received Cilostazol in combination with dual antiplatelet therapy [168]. Cilostazol in addition to aspirin and clopidogrel improved long-term outcomes after PCI in patients with ACS. In this study, ACS patients (*n* = 1212) were randomised to either standard dual antiplatelet therapy or triple therapy with addition of Cilostazol for 6 months after a successful PCI. The triple therapy group had a significantly lower incidence (10.3% versus 15.1%; HR = 0.65; *P* = 0.011) of the primary end point (cardiac death, nonfatal MI, stroke, or target vessel revascularisation at 1 year after randomisation) [212]. No significant differences were noted in the risks for major and minor bleeding. The use, however, is limited by the side effects that include headache, palpitations, and gastrointestinal disturbances.

### 19.4. Thrombin Receptor Antagonists

Thrombin is an important link in the coagulation cascade and is a potent agonist of platelet aggregation. Generation of thrombin is enhanced in DM patients and hence is a potential target for treatment. Two oral thrombin receptor antagonists are in clinical development, vorapaxar, and atopaxar [213]. Vorapaxar had an excellent safety profile when used concomitantly with aspirin and clopidogrel in a large phase II study [214]. Two large-scale phase III trials were conducted to evaluate the safety and efficacy of vorapaxar, the Trial to Assess the Effects of vorapaxar in Preventing Heart Attack and Stroke in Patients with Atherosclerosis (TRA-2P) in atherosclerosis patients and the Trial to Assess the Effects of vorapaxar in Preventing Heart Attack and Stroke in patients with ACS (TRACER) in ACS patients. An increase in intracranial haemorrhage in patients with stroke was observed in these trials. Hence TRACER trial was discontinued earlier this year and the accumulated data are yet to be presented. In TRA-2P trial, the study drug is being given only to patients with MI and peripheral vascular disease. The study drug has been withdrawn or not being given to those with a history of stroke or who have suffered a stroke during the course of the trial. Atopaxar has also shown promising results from phase II trial [215]. Further studies with these agents will provide an insight in to their use in the future.

## 20. Newer Oral Anticoagulants

In DM patients, the enhanced atherothrombotic risk is not only due to platelet reactivity but also due to dysregulation of coagulation processes. These include increased plasma coagulation factors (Factor VII and thrombin), TF, PAI-1, and decreased endogenous anticoagulants (Protein C and thrombomodulin). Several new oral anticoagulants, including antifactor IIa (Dabigatran) and antifactor Xa (Rivaroxaban, apixaban) agents, are currently being tested for long-term use in ACS patients as an adjunct to dual antiplatelet therapy, in which DM patients represent a cohort of special interest [216, 217].

## 21. Management of Endothelial Dysfunction in DM

In spite of the predominant role played by oxidative stress in DM and atherosclerosis, antioxidant therapy had no benefit in large randomised control trials [218–220]. ACEIs and angiotensin receptor blockers (ARB's) decrease superoxide production by NADPH oxidase and hence are beneficial in DM patients. Statin therapy improves endothelial function, reduces free radical production, and inhibits proinflammatory mechanisms in DM patients. Practical methods to monitor endothelial function in DM patients and to help in optimising therapy accordingly might be useful in the future.

## 22. Further Clinical Studies in DM

Anti-inflammatory drugs, PKC*β* inhibitors, and NF*κ*B inhibitors appear to hold great promise and clinical studies using these agents are in progress. Preliminary work on drugs that have favourable effects on mitochondrial function (mitochondrial superoxide production, biogenesis, dynamics, and autophagy) has been promising.

## 23. Conclusion

DM patients have increased atherothrombotic risk and recurrent ischaemic events following acute coronary syndrome and PCI despite being on currently recommended dual antiplatelet therapy, when compared to the nondiabetic population. This may partly be due to abnormal endothelial function, abnormal platelet haemostasis resulting in platelet hyperactivity, and dysregulation in the coagulation processes. GP IIb/IIIa inhibitors and bivalirudin have shown to improve acute outcomes in DM patients with ACS. Current dual antiplatelet therapy has proved successful in improving the outcomes in DM patients with ACS and remains the main stay of treatment for long-term secondary prevention and reduction of stent thrombosis. However, in the presence of DM, platelet activity and recurrent thrombotic events remain significantly high in spite of the currently recommended antiplatelet and antithrombotic therapies. Therefore, more potent antithrombotic therapies are warranted for this group of high risk patients. Clinical studies with novel therapies may provide important treatment alternatives in the future to tackle this thrombotic burden.

## Figures and Tables

**Table 1:** Endothelial dysfunction in diabetes mellitus.

Impaired vasodilatation due to decreased NO.
Increased vasoconstriction due to increased endothelin-1.
Increased expression of adhesion molecules VCAM-1 and ICAM-1.
Increased expression of chemotactic factors and proinflammatory cytokines.
Increased production of prothrombotic factors (PAI-1, TF, VWF, thromboxane).
Increased oxidative stress.
Increased PKC*β* activation.
Impaired mitochondrial biogenesis, fusion, and autophagy.

**Table 2:** Coagulation and Fibrinolysis Dysregulation in Diabetes Mellitus.

Impaired TF production.
Elevated F VII:c levels.
Increased thrombin generation.
High levels of IL-6 and fibrinogen.
Impaired fibrinolysis as a result of elevated PAI-1 levels.

**Table 3:** Outcomes from large-scale randomised placebo-controlled trials evaluating the efficacy of dual antiplatelet therapy with aspirin and clopidogrel versus aspirin alone in ACS/PCI patients in the overall study population and in DM patients.

Study	Scenario	Primary end point	*n *(overall)	% of events and association measure (overall)	*n *(DM)	% of events and association measure (DM)
CURE [22]	UA/NSTEMI	Cardiovascular death, nonfatal MI, or stroke at 1 year	12 562	9.3 versus 11.4	2840	14.2 versus 16.7
RR (95% CI)				0.80 (0.72–0.90)		0.84 (0.70–1.02)

PCI-CURE [159]	CURE patients undergoing PCI	Cardiovascular death, MI, or urgent TVR at 30 days	2658	4.5 versus 6.4	504	12.9 versus 16.5
RR (95% CI)				0.70 (0.50–0.97)		0.77 (0.48–1.22)

		Death, MI, or stroke at				
CREDO [158]	Elective PCI	1 year	2116	8.5 versus 11.5	560	NR
						
RRR (95% CI), %				26.9 (3.9–44.4)		11.2 (−46.8–46.2)

COMMIT [157]	Acute MI (93% STEMI)	Death, reinfarction or stroke at discharge or 28 days	45 852	9.2 versus 10.1	NR	NR
OR (95% CI)				0.91 (0.86–0.97)		

CLARITY [156]	STEMI with fibrinolysis	Occluded infarct-related artery on angiography or death or recurrent MI before angiography	3491	15.0 versus 21.7	575	NR
OR (95% CI)				0.64 (0.53–0.76)		

PCI-CLARITY [155]	CLARITY patients undergoing PCI	Cardiovascular death, recurrent MI or stroke at 30 days	1863	3.6 versus 6.2	282	6.0 versus 10.1
OR (95% CI)				0.54 (0.35–0.85)		0.61 (0.24–1.53)

CURE: Clopidogrel in Unstable angina to Prevent Recurrent Events; CREDO: Clopidogrel for Reduction of Events During Observation; COMMIT: Clopidogrel and Metoprolol in Myocardial Infarction Trial; CLARITY: Clopidogrel as Adjunctive Reperfusion Therapy; UA: Unstable Angina; TVR: Target Vessel Revascularisation; RR: Relative Risk; RRR: Relative Risk Reduction; OR: Odds Ratio; NR: Not Reported. Adapted from [74].
